# Antenna/Propagation Domain Self-Interference Cancellation (SIC) for In-Band Full-Duplex Wireless Communication Systems

**DOI:** 10.3390/s22051699

**Published:** 2022-02-22

**Authors:** Yuenian Chen, Can Ding, Yongtao Jia, Ying Liu

**Affiliations:** 1 Science and Technology on Antenna and Microwave Laboratory, Xidian University, Xi’an 710071, China; yuenianchen@stu.xidian.edu.cn (Y.C.); liuying@mail.xidian.edu.cn (Y.L.); 2 Global Big Data Technologies Centre, University of Technology Sydney, Ultimo, NSW 2007, Australia; Can.Ding@uts.edu.au

**Keywords:** antenna isolation, antenna/propagation (AP) domain, in-band full-duplex (IBFD), self-interference cancellation (SIC)

## Abstract

In-band full duplex (IBFD) is regarded as one of the most significant technologies for addressing the issue of spectrum scarcity in 5G and beyond systems. In the realization of practical IBFD systems, self-interference, i.e., the interference that the transmitter causes to the collocated receiver, poses a major challenge to antenna designers; it is a prerequisite for applying other self-interference cancellation (SIC) techniques in the analog and digital domains. In this paper, a comprehensive survey on SIC techniques in the antenna/propagation (AP) domain is provided and the pros and cons of each technique are studied. Opportunities and challenges of employing IBFD antennas in future wireless communications networks are discussed.

## 1. Introduction

As the demands for connectivity and data rates increase exponentially, there has been a shortage of frequency resources to support new systems, and this spectrum sparsity problem is becoming significantly more severe for 6G. In-band full-duplex (IBFD) technology, which allows transceivers to transmit and receive in the same frequency band simultaneously, is considered as a key solution to the problem of spectrum scarcity. By enabling the full-duplex (FD) mode, a single radio can achieve double throughput comparing to the conventional half-duplex (HD) counterpart [1,2]. IBFD is also known as simultaneously transmit and receive (STAR) systems.

**Figure 1:**
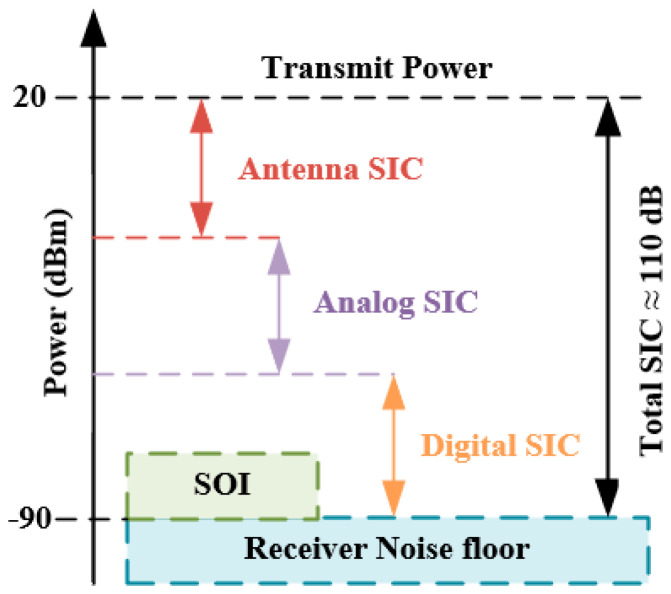
Total 110 dB self-interference cancellation (SIC) realized by SIC techniques in different domains.

A fundamental challenge to IBFD systems is self-interference (SI), which is the signal received by a receiver from a collocated transmitter [3]. SI is typically much stronger than the signal received from an intended distant transmitter. Since the SI in an IBFD system occupies the same frequency band but has much higher power than the desired signal, it must be cancelled first in order for any receiver to operate. To enable an IBFD operation, one would need to sufficiently reduce the SI to below the noise floor ideally. As Figure 1 shows, the SI power is basically determined by the transmit power and receiver noise floor and it can be anywhere between 90 and 120 dB (a billion to a trillion times) more powerful than the signal of interest (SOI) depending on the applications. For example, if the transmit power level is + 20 dBm and the thermal noise floor of the receiver is −90 dBm, the SI should be suppressed by 110 dB. This powerful SI is tremendously sensitive to the frequency selectivity, time variance, nonlinear distortion, and phase noise and these challenges are further exacerbated when integrated implementations targeting cost-sensitive and form-factor-constrained mobile devices are considered [4].

In the past decade, a long-held assumption in wireless system designs is that it is generally not possible for radios to receive and transmit on the same frequency band at the same time because of the SI [5]; and radios have to operate in HD mode (i.e., either transmit or receive, but not both simultaneously) in the same frequency band. Recently, researchers in both industry and academia have proposed various SI cancellation (SIC) techniques to enable IBFD operations [4,6,7,8], which offers the potential to complement and sustain the evolution of 5G technologies toward higher data rates and denser heterogeneous networks [9].

The goal of any SIC technique is to estimate the SI signals in the receiver in order to cancel them [10]. SIC techniques can be categorized into passive SIC and active SIC. Passive SIC refers to the SIC techniques applied in the antenna/propagation (AP) domain, which are used to electromagnetically isolate the transmitting (TX) and receiving (RX) antennas. Active SIC techniques are usually applied in the digital and analog domains to exploit the knowledge of its own transmit signal to cancel the self-interference, i.e., to generate a cancellation signal in the receive signal path to null the self-interference [11]. Note that passive SIC is a one-off design, thus it has a much lower complexity and cost than active SIC that adaptively changes with the radio environment.

**Figure 2:**
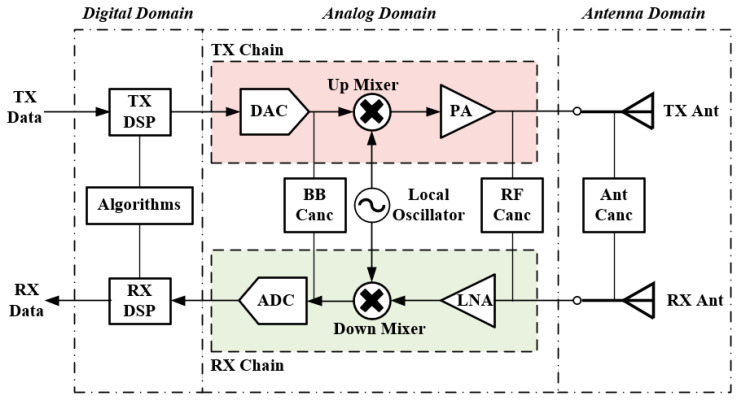
Typical in-band full-duplex (IBFD) system architecture with contents and boundaries of SIC in the digital, analog, and antenna/propagation (AP) domain.

Figure 2 illustrates the contents and boundaries of SIC in three domains in a typical IBFD system. It is clear that AP domain cancellation is the first line-of-defense against SI [11] and it is also a prerequisite for applying other SIC techniques in the analog and digital domains. Analog domain cancellation is capable of preventing the high-power SI inflicted by the analog-to-digital converter (ADC), which would desensitize the automatic gain control (AGC) owing to signal leakages [12]. This suppression may occur either before or after the downconverter and the low-noise amplifier (LNA). Digital domain cancellation techniques aim to cancel SI after the ADC by applying sophisticated digital signal processing (DSP) techniques to the received signal [3]. In a practical and efficient IBFD system, SIC in all domains is indispensable to achieve the required 110 dB total SIC, as is shown in Figure 1. Note that the better the cancellation achieved in the AP domain, the less the pressure and difficulty will be suffered in the rest of the domains. Since the AP domain SIC has a much lower cost and complexity compared to the SIC in the digital and analog domains, the cost of IBFD system could be notably reduced if higher isolation can be achieved in the AP domain.

IBFD communication has widespread applications in the wireless realm. For civilian applications, it is considered for wireless local area network (WLAN) systems [13], radio frequency identification (RFID) readers [14], vehicle-to-vehicle (V2V) communications [15], wireless data and power transmission systems [16], etc. Besides, combined with relaying and multiple-input-multiple-output (MIMO) technologies, IBFD relay systems [17,18] and MIMO systems [7,19], which further improve the capacity of systems, are appropriate candidates for solving data congestion in wireless communication in 5G and beyond. IBFD is also in high demand in military applications such as electronic warfare systems, where it offers the possibility to monitor weak signals and simultaneously introduce jamming signals in the channel to improve security [20,21].

In this paper, we survey and compare various reported SIC techniques in the AP domain that have a potential to significantly reduce the cost of IBFD systems. They are categorized according to the different working mechanisms and the applicability in different environment of each kind of SIC technique is also discussed. Although there are some reviews on IBFD in the literature [3,9,12,18,22,23], the antenna technologies that facilitate the realization of IBFD were not thoroughly studied. This paper, from the antenna’s perspective, elaborates the design considerations and compares the pros and cons of each antenna technique. It also pointed out some opportunities and challenges for antenna researchers to better contribute to the improvement of future IBFD systems.

## 2. Passive Self-Interference Cancellation

Passive SIC was initially realized by antenna separation only and was defined in [16] as the signal power attenuation imposed by the path loss between the TX and RX antennas of the same device. However, passive SIC has evolved substantially in recent years and physical separation is the most fundamental approach among various available techniques. In this paper, passive SIC is summarized as techniques embedded on antennas or their feed networks to electromagnetically isolate the TX and RX antennas. 

**Figure 3:**
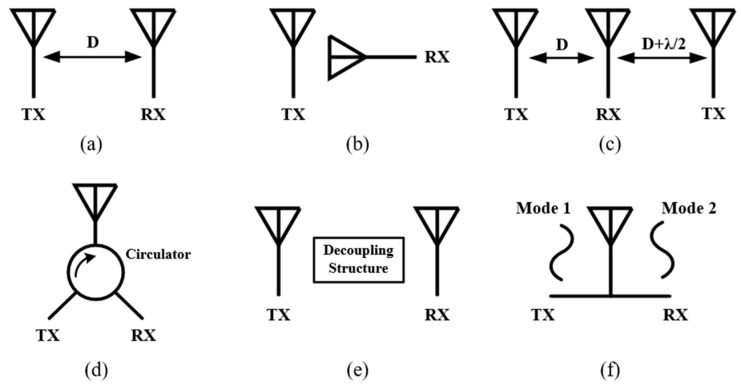
Passive SIC techniques in the AP domain. (**a**) Antenna separation. (**b**) Polarization orthogonality. (**c**) Near-field cancellation. (**d**) Isolation feed network. (**e**) Decoupling surface/structure. (**f**) Orthogonal antenna modes.

According to the different working mechanisms, the reported SIC techniques can be divided into six categories as illustrated in Figure 3. On the other hand, they can be further classified into single/shared antenna or multi-antenna systems, depending on whether the system use the same or different antennas for transmitting and receiving.

### 2.1. Antenna Separation

**Figure 4:**
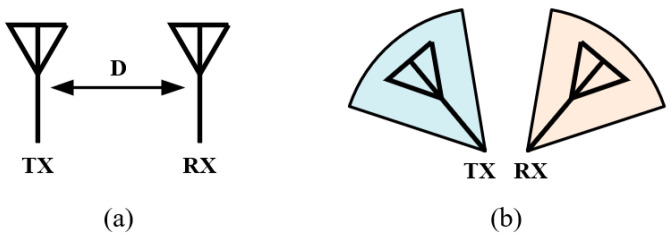
Two types of antenna separation. (**a**) Spatial separation. (**b**) Directional separation.

As shown in Figure 4a, physically separate the TX and RX antennas is the earliest and easiest method to reduce SI. The electromagnetic filed attenuation between the two antennas, namely the path loss, can be calculated by:

(1) $${L=20\log}_{10}(\frac{4\pi d}{\lambda })$$

where *L* is the path loss in decibels, *d* is the distance between the TX and RX antennas, and *λ* is the wavelength in free space [24]. Although increasing the distance can surely improve the isolation between the antennas, the merit is limited, e.g., the path loss is only increased by 6 dB when the distance is doubled. Another more efficient method to “separate” the TX and RX antennas is to direct their radiation beams into different directions (directional separation), as shown in Figure 4b, leading to a weaker coupling despite the relatively closer distance between them.

**Figure 5:**
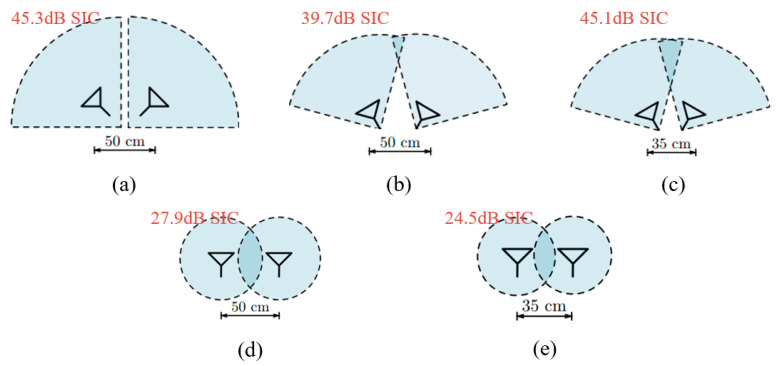
Five configurations of antenna separation with their SIC performance investigated in [11]. (**a**) 90° directional separation with 50 cm spatial separation. (**b**) 60° beam separation with 50 cm antenna separation. (**c**) 60° beam separation with 35 cm antenna separation. (**d**) 50 cm antenna separation. (**e**) 35 cm antenna separation. Note the antennas in (**a**–**c**) have 90° beamwidth and the antennas in (**d**–**e**) are omnidirectional.

In [11], these two kinds of antenna separation techniques are tested in both an anechoic chamber and a reflective room to evaluate their performance and contribution to SIC. Figure 5 shows the five configurations together with their SIC performance in the anechoic chamber. As expected, increasing the separation distance leads to a better isolation, but the incremental is very limited, e.g., the SIC improvement is only around 3 dB by increasing the distance from 35 cm to 50 cm (see Figure 5d,e). On the other hand, the directional separation proves to be more effective than the spatial separation. It also should be noted that these two separation techniques can be applied together to realize better SIC [25,26].

Antenna separation, either the spatial one or directional one, is easy to implement but has many restrictions. Spatial separation is not preferable when the size is a major concern since a better isolation is obtained by increasing the distance between the TX and RX antennas, which inevitably leads to a larger size of the antenna system; directional separation is definitely not an option when the TX and RX antennas are required to point to the same direction or when omnidirectional broadcasting is needed. Moreover, antenna separation is only applicable for multi-antenna systems. The good side is that this method can be easily blended with other techniques to attain better performance [27,28].

### 2.2. Polarization Orthogonality

Two electromagnetic waves with orthogonal polarizations are naturally isolated from each other. Therefore, SIC can be achieved by transmitting in one polarization and receiving in its orthogonal polarization. The orthogonal polarizations could be two linear polarizations or a pair of circular polarizations. 

**Figure 6:**
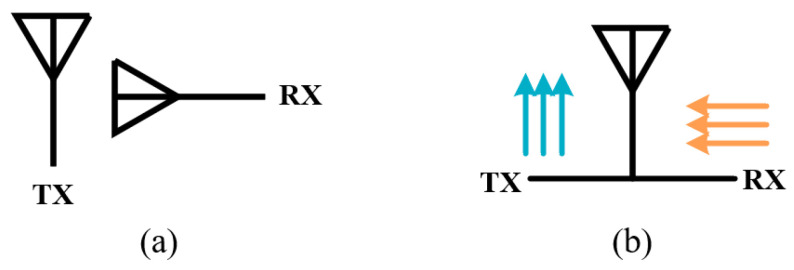
Polarization orthogonality realizations in (**a**) multi-antenna system and (**b**) single/shared antenna system.

As shown in Figure 6, polarization orthogonality can be realized either on two or more antennas having orthogonal polarizations or only one dual-orthogonal-polarized antenna. For example, in [29], a cone antenna having vertical polarization is used for transmitting and four dipole antennas having horizontal polarization are arranged around the cone antenna for receiving. The isolation between the two polarizations is > 37 dB within the operation band. Good SIC could be also achieved on one antenna by exciting two orthogonal linear polarizations of patch antenna [30,31,32,33], [34,35], horn antenna [36], cone antenna [29], spiral antenna [37], slot antenna [38,39] or dipole antenna [40,41,42].

**Figure 7:**
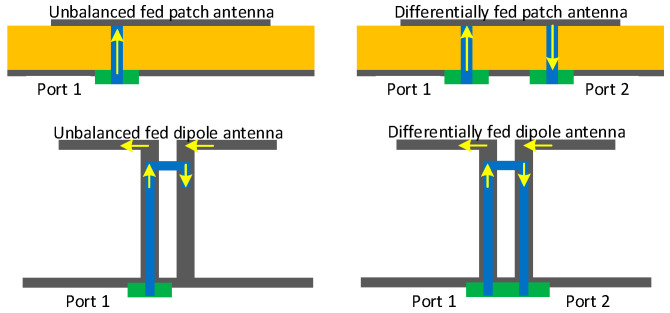
Illustration of unbalanced fed and differentially fed patch and dipole antennas.

**Figure 8:**
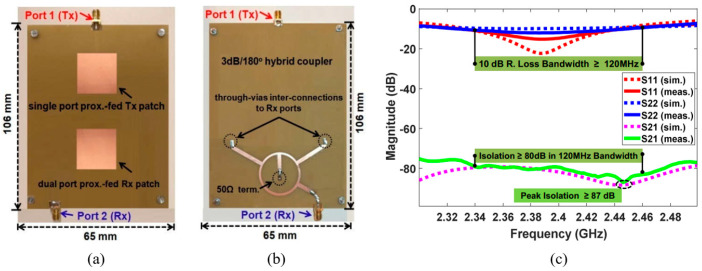
Proposed IBFD antenna with state-of-the-art SIC performance in [54]. (**a**) Antenna elements (top view). (**b**) Ring coupler (bottom view). (**c**) Reflection and transmission coefficients (S parameters).

Ideally, the isolation between two polarizations that are perpendicular to each other is infinite. However, in reality, antennas always have some cross-polarization radiation, which leads to unneglectable couplings between the orthogonal polarizations [43]. As shown in Figure 7, compared to the more widely used unbalanced feeding, differentially feed (balanced feed) an antenna can attain higher polarization purity with a cost of additional feed port. Isolation between two polarizations can be substantially increased by applying differential feed to either one of the TX/RX ports [44,45] or both the two ports [46,47,48]. In [17,49,50,51,52], by employing power dividers [53] with 180° phase shift or hybrid couplers in the feed networks, the SI is further attenuated because they create two coupling paths with out-of-phase signals that cancel with each other to some extent. As shown in Figure 8, two patch antennas with orthogonal polarizations are designed in [54], i.e., one is fed unbalanced and the other is fed differentially with a 3 dB coupler, leading to an ultra-high SIC of 80 dB in the AP domain.

To provide an intuitive understanding of the capability of this method, Table 1 summarizes some state-of-the-art IBFD antennas based on polarization orthogonality. One upper hand of this approach is the relatively smaller size as the TX and RX antennas share the same aperture. As one of the most commonly used passive SIC techniques, polarization orthogonality can be easily realized with simple configurations, e.g., patch and dipole antennas. With additional complexity, differential feeding techniques can be used to feed dual-polarized antenna/s to achieving higher level of SIC. Another advantage is that this technique is suitable for both single-antenna and multi-antenna systems, which offers additional degree of freedom in IBFD systems. However, the polarization purity of antenna is very sensitive to the antenna’s geometrical symmetry and external electromagnetic environment. Therefore, when used in practice, the SIC performance achieved using this method may not be as good as predicted in the laboratory.

### 2.3. Near-Field Cancellation

The idea of near-field cancellation or antenna cancellation [6] is to allocate two or more TX (or RX) antennas around a RX (or TX) antenna to make the TX (or RX) signals cancel at the RX (or TX) port. This kind of cancellation can be realized in several means. 

**Figure 9:**
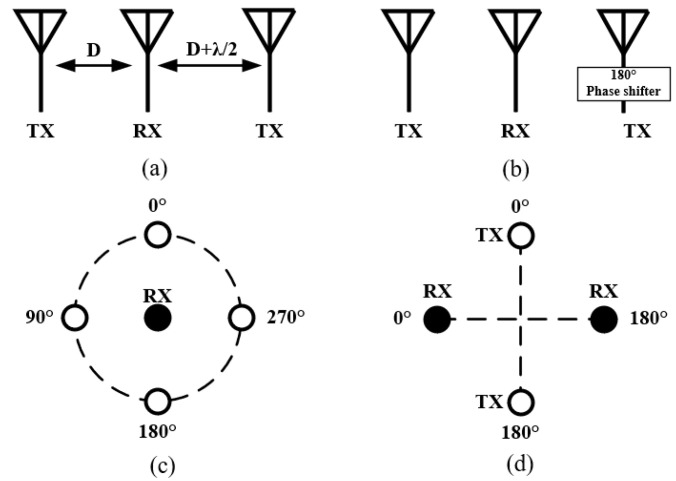
Near-field cancellation in four configurations. (**a**) λ/2 distance difference between transmitting (TX) antennas. (**b**) 180°phase difference between TXs. (**c**) Circular TX array with gradient phases. (**d**) TX and receiving (RX) antenna pairs with differential feeding.

**Figure 10:**
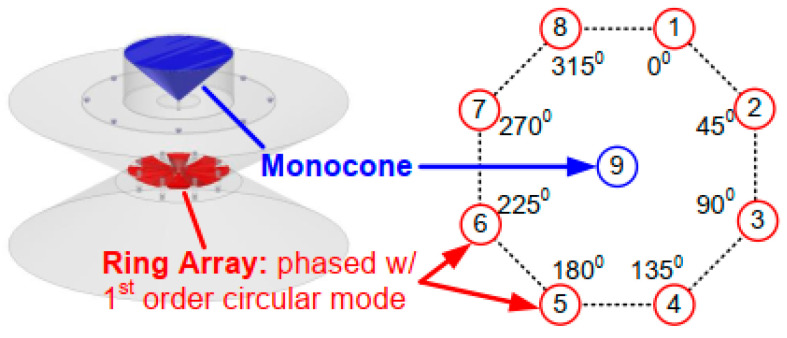
Near-field cancellation realization in [60].

As shown in Figure 9a,b, the cancellation can be achieved by placing two TX antennas at the distance of D and D + λ/2 away from the RX antenna, or by feeding two TX antennas with 180° phase difference at the same distance away from the RX antenna [55,56]. As illustrated in Figure 9c, one can also acquire near-field cancellation by circularly arranging several TX antennas around a RX antenna and exciting the circular TX antenna array with gradient phases [13,15,57,58,59,60,61]. In this configuration, the center antenna is usually cone or monopole antenna and the circularly-arranged antennas are often monopole or dipole antennas, as shown in Figure 10, which are often used to achieve omnidirectional radiation to ensure coverage.

As shown in Figure 9d, near-field cancellation can also be achieved by using two pairs of circular-polarized antenna elements [62]. Because of the anti-phase input signal of the two differential feed ports, there exists a near-field radiation null at the symmetric axis of the two ports of a differentially fed antenna. By placing the RX (TX) antenna at the symmetric axis of the TX (RX) antenna, near-field cancellation can be realized. 

Except the one shown in Figure 9a, near-field cancellation attained by the strategies illustrated in Figure 9b,d ideally has a wide operation band since the achieved symmetry for SIC is independent of frequency. However, the performance of this cancellation can be greatly deteriorated by the errors in the magnitudes and phases of the input signals. Therefore, it has a strict requirement on the accuracy of the employed phase shifters and power dividers. In addition, it is worth to mention that near-field cancellation can only be applied on multi-antenna systems thus the size is larger and the complexity and difficulty in the design of the feeding networks are critical. Table 2 lists the state-of-the-art IBFD antenna systems based on near-field cancellation strategies. As expected, the achieved maximum SIC using this method is not super high (<50 dB), but the working bandwidth can be very wide, e.g., from 0.96 to 8.2 GHz in [60].

### 2.4. Isolation Feed Network

**Figure 11:**
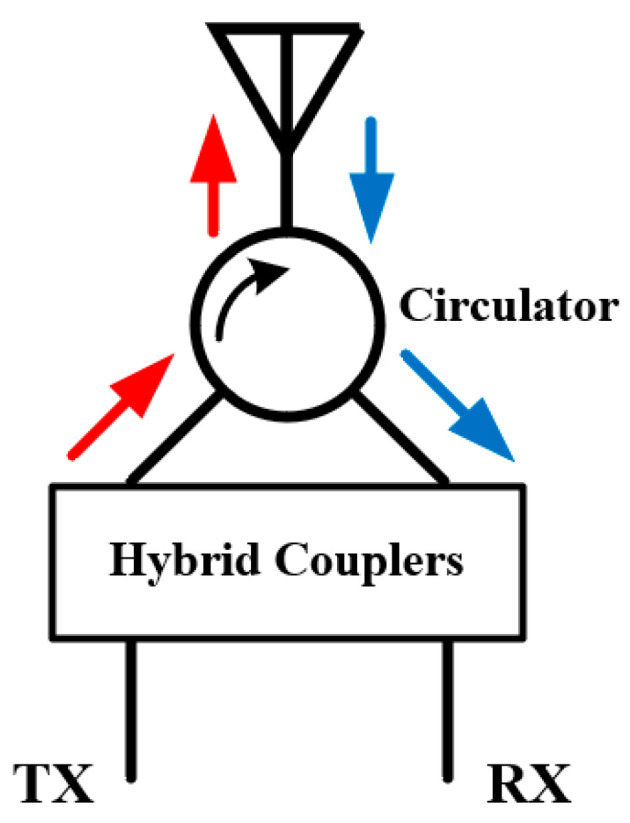
Typical isolation feed network employing circulators and couplers.

When only a single antenna is available in the communication system, a circulator, in which the input signal only flows out the port that after the input one as shown in Figure 11, can be implemented in its feed network to separate two isolated signal paths (one for transmitting and the other for receiving) as shown in Figure 11. This method is usually combined with a properly designed hybrid network to deal with the signal leakage of circulator and reflection due to the antenna mismatch, which may deteriorate the SIC performance.

**Figure 12:**
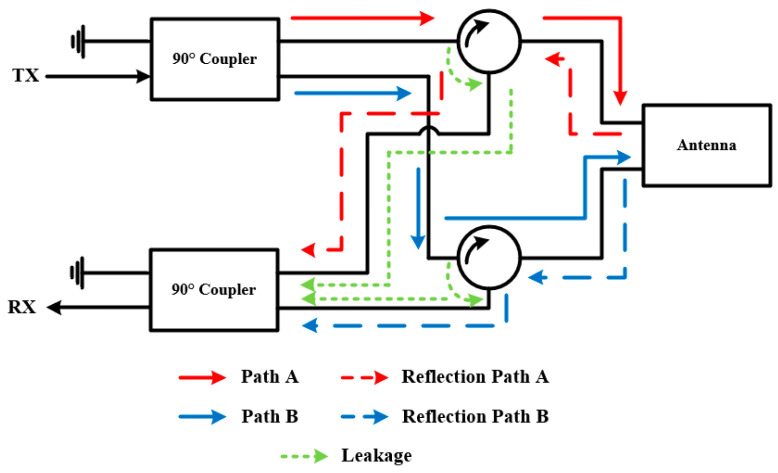
Typical isolation feed network employing circulators and couplers.

As an example, Figure 12 shows the isolation feed network proposed in [63] to achieve IBFD communication. The 90° coupler connected to the TX port, which is able to provide ± 90° phase difference between two output ports, will separate the TX signal into two signal paths, i.e., path A and path B with 90° phase difference. In ideal conditions, all the input signals will enter the antenna as shown by the red and blue solid lines. However, in practice, the refection signal caused by antenna mismatching (as shown by the red and blue dash lines) and the leakage signals (as shown by the green dot lines) of circulators cannot be neglected. These signals will reach the RX port through another 90° coupler, which makes a total 180° phase difference for the two reflection signals and the two leakage signals. They will eventually cancel out at the RX port, making the isolation between the two ports remains at a high level.

To demonstrate the capability of this method, Table 3 summarizes all the comparable designs [64,65,66,67,68,69,70], whose isolation is attained with the combination of 180° or 90° couplers and circulators. This technique is able to create two well isolated signal paths for a single antenna by using circulators and hybrids. However, the imperfection of the available devices limits its performance. For example, in [71], with ideal circulators, the resultant isolation is >80 dB, but the measurement results show that only >30 dB isolation is achieved when using practical components. Although the hybrid networks can help to alleviate this issue by cancelling the reflections and leakage to some extent, it requires a high symmetry of the network and uniformity of the employed devices. For example, if the two circulators shown in Figure 12 have different leakage and reflection ratios, the symmetry of the network is degraded, so as the performance. Another drawback of this technique is the fact that, although the antenna itself could be small, the size of the isolation feed network is inevitably larger and the employed additional components can lead to higher cost of the whole system. Besides, the complicated feed network might introduce extra insertion loss, leading to deteriorated antenna efficiency.

### 2.5. Decoupling Structure

**Figure 13:**
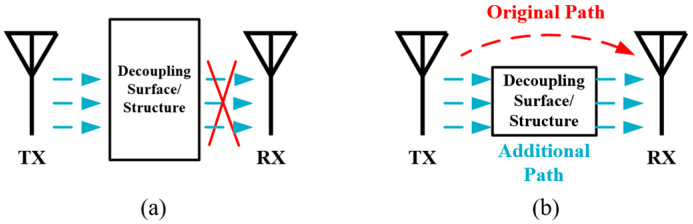
Two kinds of decoupling structure. (**a**) Filter. (**b**) Additional coupling path.

There has been intensive research for many years to reduce antenna decoupling. Among the available methods, decoupling structures that are placed between two closely spaced antennas serve as good candidates for IBFD antenna systems. As illustrated in Figure 13a, one kind of the decoupling structure acts as a band-stop filter, which can block the coupling wave at the operation bandwidth. Examples based on this mechanism include defected ground structure (DGS) [72], wave trap structure (WTS) [73,74], high impedance surface (HIS) [75,76], frequency selective surface (FSS) [77,78], electromagnetic band gap (EBG) [79,80] and so on. The other kind of decoupling structures like resonant baffles [81,82], antenna decoupling surface (ADS) [83] and neutralization line (NL) [84,85] can introduce an additional coupling path whose phase is set opposite to the original coupling path, thus the coupling of two paths cancels out, as shown in Figure 13b. In addition, some studies [11,86] prove that electromagnetic absorbing materials, which are basically composed of dielectric substrate with high loss tangents, are also able to improve the isolation by absorbing the coupling energy.

Table 4 lists and compares some decoupling structures that have a potential to reduce the SI in IBFD systems. Thanks to these decoupling techniques, the isolation can be improved to a great level. The pros of this kind of methods come from its numerous manifestations that offers a great design flexibility, and the cons are that they usually pose negative effects on the antennas’ radiation patterns. Note that this approach also employs multiple antennas but the size of the employed antenna is usually smaller than those of the spatial separation and near-field cancellation techniques. This is because the TX and RX antennas can be placed closer to each other thanks to the decoupling structures.

### 2.6. Orthogonal Antenna Modes

**Figure 14:**
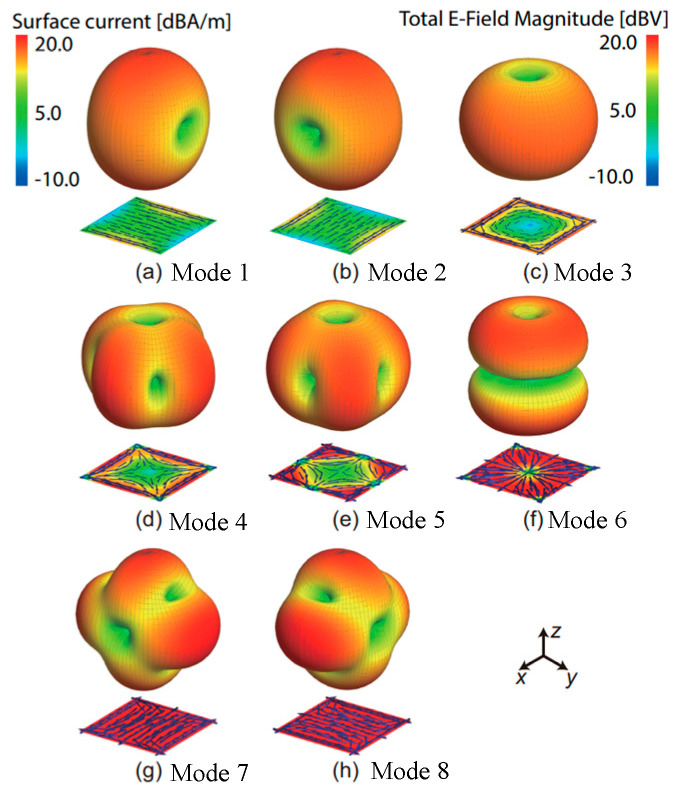
Radiation patterns of seven characteristic modes of a patch antenna in [87].

At last, there are also a few examples of achieving SIC by exciting a pair of orthogonal characteristic modes on one antenna with different feed positions. For example, as shown in Figure 14, reference [87] presents the current distributions and radiation patterns of seven modes of a patch antenna. By exciting the two dominant characteristic modes with orthogonal polarizations, i.e., mode 1 and mode 2, an isolation of more than 30 dB between the two modes is achieved. It is also proved capable of exciting two characteristic modes with the same polarization, i.e., mode 2 and mode 7, to attain an isolation of 58 dB at 2.4 GHz. However, it’s worth to mention that the radiation patterns of the two modes are different. This technique is also used in chassis-mode mobile MIMO antennas to excite two orthogonal characteristic modes, thus improving the isolation to about 21 dB in [88]. Besides, in [89], the microstrip-coupled coplanar waveguide (CPW) is used at the TX port to excite a stepped-slot antenna in the CPW odd mode. On the opposite side of the antenna, a microstrip T-junction power divider is employed at the RX port to feed two offset-fed stepped-slot antennas in even mode, achieving more than 50 dB isolation. In [90], by exploring a shared aperture cavity-like structure with two highly orthogonal modes (quarter-wavelength slot mode and half-mode cavity mode), high isolation of 43 dB between TX and RX ports is achieved. In recent IBFD designs [91,92], the technique based on common-mode and differential-mode cancellation is also proved feasible to realize high isolations between co-polarized antennas.

Although this method offers good isolation on a single antenna at certain circumstances, it is not a mature method and is not easy to implement. It takes quite some efforts to select and excite the right characteristic modes, which increases the design complexity. Besides, when exciting high-order modes, the operation bandwidth is usually narrow. The sizes of the employed antennas are usually small as it only needs single antenna and no additional feed network is required.

At last, the aforementioned strategies are summarized and compared in Table 5 to provide a reference manual for antenna designers to select the most suitable method according to the practical scenarios.

## 3. Opportunity and Challenges

The advances in antenna technology provide many powerful means to address the SI in the AP domain. The efforts in the AP domain can significantly alleviate the cost and design complexity in the analog and digital domains, thus greatly reducing the cost of the entire IBFD system. How to utilize the antenna technology to maximizing the capability of SIC provides many opportunities but also have many challenges.

### 3.1. Combination of Multiple SIC Techniques in the AP Domain

As surveyed in Section 2, there are plenty of effective SIC techniques in the AP domain and each technique inherently has particular pros and cons. To overcome their limitations, several different techniques can be blended and implemented on one design. In achieving splendid SIC performance, the key is to correctly identify the different causes of the SI and address each one differently but systematically. For example, in [93], the proposed antenna array is comprised of four dual-circularly-polarized antennas (two for TX and the other two for RX). More than 38 dB isolations between the TX and RX antennas, between the two TX antennas, between the two RX antennas, and between the two polarizations of each antenna are achieved using different methods. On the other hand, a combination of different techniques is also able to increase one kind of isolation, such as the isolation between two polarizations, which leads to a final 70 dB isolation in [94]. There are already some successful examples of fusing different techniques to attain high SIC and it is anticipated that this will become a big trend in future.

### 3.2. Adaptive/Tunable SIC Techniques in the AP Domain 

**Figure 15:**
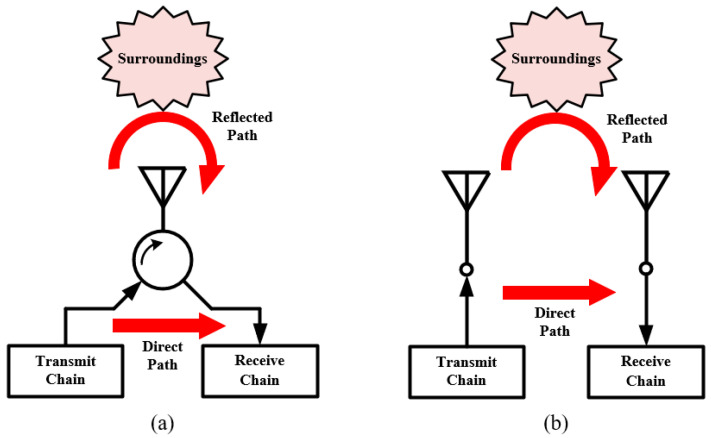
Two types of self-interference in IBFD communication with (**a**) single/shared antenna and (**b**) multiple antennas.

As illustrated in Figure 15, the SI generally comes from two paths. One is direct path, which refers to the direct interference between the TX and RX chains and the other is caused by the near surroundings, namely reflected path. The passive SIC methods studies in Section 2 can only suppress the SI of direct path. When the designed antennas are used in practical environment, the SI of reflected path caused by nearby surroundings will surely deteriorate the system’s performance.

Some researchers [11,39,81] have proposed tunable or reconfigurable SIC techniques, which are able to address the SI of reflected path. However, these techniques have limited working states and they cannot deal with all conditions in practice. Reconfigurable antennas have emerged as a promising solution and attracted considerable attentions for the past 20 years [95,96,97]. Endowing the SIC in the AP domain with reconfigurability could be beneficial and the challenge comes from balancing its capability and complexity.

### 3.3. SIC for MIMO Antenna Systems

As a central technology of 5G, multiple-input and multiple-output (MIMO) uses multiple TX and RX antennas to exploit multipath propagation, which is able to multiply the capacity of a radio link [98]. IBFD can be combined with MIMO to further enhance the spectral efficiency. However, as both the two techniques need to reduce the couplings between the antenna elements, this brings a significant challenge to antenna designers to cope with increased number of TX/RX chains. Simply replicating the SIC designs used in single-input-single-output (SISO) antenna systems into MIMO system would not work because of the cross-talk, i.e., the interferences among different chains. Consequently, to empower practical IBFD MIMO system, the cross-talk needs to be carefully addressed and comprehensive SIC techniques for MIMO systems are needed.

### 3.4. IBFD Antenna with Two Polarizations in Both TX and RX Modes

**Figure 16:**
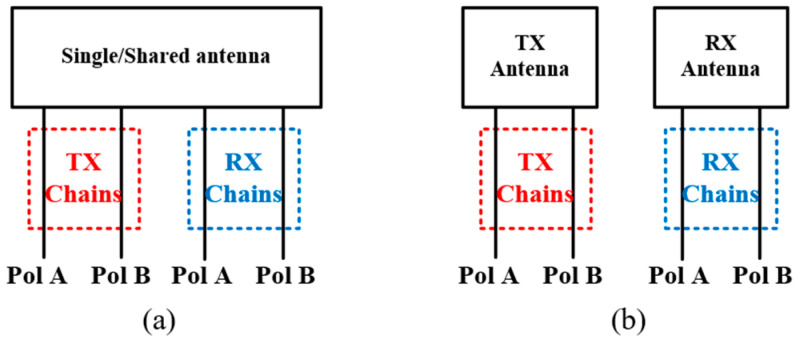
Two kinds of IBFD antenna with two polarizations in TX and RX chains. (**a**) Single/Shared antenna. (**b**) Multiple antennas.

Present IBFD antenna systems are mainly in two forms. One focuses on single/shared antenna structure with different polarizations for TX/RX chain, as shown in Figure 6b. The two polarizations are orthogonal to each other so that good SIC performance can be achieved. The other one is to use separate TX and RX antennas with same or different polarization (as shown in Figure 6a) and utilize SIC techniques to improve the isolation between them. Both the two forms have two chains (one for TX and the other for RX) and they are proved feasible and effective in IBFD systems. It is expected that the two forms can be combined to create four isolated chains in the AP domain (two for TX and two for RX, as shown in Figure 16), which makes a full utilization of the antennas and increases the flexibility of the system. Moreover, it can reduce the space occupied by the employed antennas. To make it happen, one needs to reduce the couplings between the two polarizations of the same antenna and between the antennas with same polarizations. They need to be addressed simultaneously but differently.

## 4. Conclusions

With effective SIC techniques, IBFD wireless communication systems will offer tremendous opportunities of improving spectrum efficiency in 5G and beyond. When designing an IBFD communication system or radio, one should carefully select suitable passive SIC techniques according to the number of antennas, antenna configuration, application scenarios and other related factors to achieve satisfactory SIC performance in the AP domain. As the first defense barrier of SI in the IBFD system, improving the SIC performance in the AP domain will greatly alleviate the design difficulty of the subsequent SIC in the analog and digital domains, thus substantially reducing the cost of the entire system. There is still a lot of work to be done to make a full use of the advancing antenna technologies to maximize the capability of the SIC in the AP domain, such as adaptive SIC techniques, full utilization of two orthogonal polarizations, integration with MIMO system, etc.

## Figures and Tables

**Table 1:** A comparison of IBFD antennas with polarization orthogonality.

Ref.	Antenna Type	FD Antenna Type	Feed Type	Polarization	Bandwidth (−10 dB) (FBW)	Isolation (dB)
[13]	Patch	Shared antenna	UF	Two LPs	50 MHz @ 2.4 GHz (2%)	>55
[36]	Horn	Shared antenna	UF	Two LPs	1.7–2.7 GHz (45.5%)	>45
[29]	1 Cone + 4 Dipoles	Multiple antennas	UF	Two LPs	0.8–2.7 GHz (−8.5 dB) (108.6%)	>37
[40]	Dipole	Shared antenna	UF	Two LPs	1.63–3.71 GHz (77.9%)	>28
[34]	2 Patches	Multiple antennas	UF	Two LPs	400 MHz @ 4.6GHz (8.7%)	>50
[14]	Patch	Shared antenna		Two CPs	860–940 MHz (−18 dB) (8.9%)	>25
[37]	2 Spirals	Multiple antennas	UF	Two CPs	2.5–4.7 GHz (61.1%)	>21.5
[44]	Patch	Shared antenna	UF; DF	Two LPs	50 MHz @ 2.4 GHz (2%)	>70
[54]	2 Patches	Multiple antennas	UF; DF	Two LPs	120 MHz @ 2.4 GHz (5%)	>80
[49]	2 Patches	Multiple antennas	UF; DF	Two LPs	110 MHz @ 2.5 GHz (4.4%)	>64
[46]	Patch	Shared antenna	DF	Two LPs	50 MHz @ 2.4 GHz (2%)	>72
[38]	Slot	Shared antenna	DF	Two LPs	93.4–95.6 GHz (2.3%)	>55
[48]	Patch	Shared antenna	DF	Two LPs	2.2–2.5 GHz (12.8%)	>40

FBW: fractional bandwidth; UF: unbalanced feed; DF: differential feed; LP: linear polarization; CP: circular polarization.

**Table 2:** A comparison of IBFD antennas based on near-field cancellation.

Ref.	NFC Type	Number of Antennas	Antenna Spacing	Bandwidth (−10 dB) (FBW)	Gain (dBi)	Isolation (dB)
[6]	λ/2 distance difference	TX-2; RX-1	D; D+λ/2	5 MHz @ 2.48 GHz (0.2%)	-	~30 dB
[55]	180° phase difference	TX-1; RX-2	λ_0_/4	2.435–2.51 GHz (3%)	<3.4 (TX); <6.4 (RX)	>47
[57]	Circular array	TX-1; RX-4	0.44 λ_0_	2.4–2.7 GHz (11.8%)	<3.2	>38
[13]	Circular array	TX-4; RX-1	0.38 λ_0_	2.33–2.85 GHz (20.1%)	<3.6 (TX); <0.6 (RX)	>40
[58]	Circular array	TX-4; RX-1	-	3.1–3.6 GHz (14.9%)	-	>50
[59]	Circular array	TX-8; RX-1	-	2.4–2.5 GHz (4.1%)	-2	>50
[60]	Circular array	TX-1; RX-8	-	0.96–8.2 GHz (158.1%)	-	>50
[61]	Circular array	TX-1; RX-4	0.77 λ_0_	0.6–1.75 GHz (97.9%)	-	>50
[15]	Circular array	TX-8; RX-1	-	60 MHz @ 2.45 GHz (2.4%)	-	>53
[62]	TX and RX pairs	TX-2; RX-2	~ λ_0_	6–7.2 GHz (18.2%)	>8.7	>40

**Table 3:** A comparison of IBFD antennas with isolation feed network.

Ref.	IFN Configuration	Polarizations	Insertion Loss (dB)	Bandwidth (−10 dB) (FBW)	Gain (dBi)	Isolation (dB)
[63]	2 90° hybrids; 2 circulators	Same CP of TX and RX	0.75	902–928 MHz (2.9%)	-	>40
[65]	2 180° hybrids	Same CP of TX and RX	-	0.5–3.5 GHz (150%)	>3	>37
[66]	2 90° hybrids; 2 180° hybrids; 2 circulators	Same CP of TX and RX	0.35 (circulator)	4–8 GHz (66.7%)	>1	>30
[67]	2 90° hybrids; 4 180° hybrids	TX: RHCP; RX: LHCP	-	4–8 GHz (66.7%)	>7	61 (average)
[64]	2 90° hybrids; 4 180° hybrids; 4 circulators	Same CP of TX and RX	-	0.5–2.5 GHz (133.3%)	-	>40
[68]	2 90° hybrids; 4 180° hybrids	TX: RHCP; RX: LHCP	-	2.4–2.5 GHz (−22 dB) (4.1%)	~7	>47
[69]	1 90° hybrids; 1 power divider; 2 180° hybrids	-	-	1.75–1.85 GHz (5.6%)	-	>30
[71]	2 90° hybrids; 5 180° hybrids; 4 circulators	TX: RHCP/LHCP; RX: RHCP/LHCP	-	2–8 GHz (120%)	>3	>27
[70]	2 90° hybrids; 4 180° hybrids	TX: RHCP/LHCP; RX: RHCP/LHCP	-	0.8–3 GHz (115.8%)	TX: >10 RX: >5	>40

**Table 4:** A comparison of antennas with different types of decoupling structure.

Ref.	Decoupling Structure	Spacing	Bandwidth (−10 dB) (FBW)	Gain (dBi)	Isolation (dB)
[72]	DGS	-	770 MHz @ 3.2 GHz (24.1%)	<4	>36
[73,74]	WTS	~0.9λ	222 MHz @ 2.6 GHz (−6 dB) (8.5%)	>9	>60
[75]	HIS	4λ	6–19 GHz (104%)	>7	>60
[76]	HIS	-	2.04–2.06 GHz (1%)	-	>45
[78]	FSS	0.5λ	28–34 GHz (19.4%)	-	>20
[79]	EBG	0.5λ	125 MHz @ 5 GHz (2.5%)	-	>30
[81]	Baffles	-	3.3 GHz	-	>60
[83]	ADS	~0.6λ	3.3–3.8 GHz (14.1%)	~9	~25
[84]	NL	~0.06λ	3.1–5 GHz (46.9%)	~3	>22

**Table 5:** Comparison of different IBFD SIC techniques in the AP domain.

SIC Techniques	Antenna Number	Antenna Size	Advantages	Disadvantages
**Antenna separation**	Multiple antennas	Large	(1) Easy to implement (2) Easy to integrate with other techniques	Restrictions on antenna radiation pattern (for directional separation only)
**Polarization orthogonality**	Single/shared antenna; Multiple antennas	Small	(1) Easy to implement (2) High performance of SIC (3) Good radiation performance	(1) Sensitive to the symmetry of the antenna structure and the imperfection of feed network
**Near-field cancellation**	Multiple antennas	Large	(1) Wide bandwidth (2) High performance of SIC	(1) High complexity of feed network (2) Sensitive to the imperfection of feed network 3) Additional insertion loss.
**Isolation feed network**	Single/shared antenna; Multiple antennas	Small antenna, large feed network	(1) Simple antenna configuration (2) It transmit and receive signals using the same polarization, so it can be combined with polarization orthogonality to generate more isolated signal paths	(1) Extra components in the feed network (2) Sensitive to the performance of circulators and hybrids
**Decoupling surface/structure**	Multiple antennas	Medium	(1) Various designs available for different application scenarios (2) Reconfigurability	(1) Complex antenna configuration (2) Narrow bandwidth (3) Probable negative effects on radiation pattern
**Orthogonal antenna modes**	Single/shared antenna; Multiple antennas	Small	Flexible choice of radiation patterns and polarizations of antennas	(1) High complexity of antenna design and configuration (2) Narrow bandwidth

## Data Availability

Not applicable.
